# Recent Developments in Small-Molecule Ligands of Medicinal Relevance for Harnessing the Anticancer Potential of G-Quadruplexes

**DOI:** 10.3390/molecules26040841

**Published:** 2021-02-05

**Authors:** Loukiani Savva, Savvas N. Georgiades

**Affiliations:** Department of Chemistry, University of Cyprus, 1 Panepistimiou Avenue, 2109 Nicosia, Cyprus; savva.loukiani@ucy.ac.cy

**Keywords:** G-quadruplex, telomerase inhibition, proto-oncogene promoters, ribosomal DNA, anticancer agents, small-molecule ligands

## Abstract

G-quadruplexes, a family of tetraplex helical nucleic acid topologies, have emerged in recent years as novel targets, with untapped potential for anticancer research. Their potential stems from the fact that G-quadruplexes occur in functionally-important regions of the human genome, such as the telomere tandem sequences, several proto-oncogene promoters, other regulatory regions and sequences of DNA (e.g., rDNA), as well as in mRNAs encoding for proteins with roles in tumorigenesis. Modulation of G-quadruplexes, via interaction with high-affinity ligands, leads to their stabilization, with numerous observed anticancer effects. Despite the fact that only a few lead compounds for G-quadruplex modulation have progressed to clinical trials so far, recent advancements in the field now create conditions that foster further development of drug candidates. This review highlights biological processes through which G-quadruplexes can exert their anticancer effects and describes, via selected case studies, progress of the last few years on the development of efficient and drug-like G-quadruplex-targeted ligands, intended to harness the anticancer potential offered by G-quadruplexes. The review finally provides a critical discussion of perceived challenges and limitations that have previously hampered the progression of G-quadruplex-targeted lead compounds to clinical trials, concluding with an optimistic future outlook.

## 1. Introduction

G-quadruplexes, a family of tetraplex helices, are non-canonical secondary structures derived from guanine (G)-rich sequences of nucleic acids and exhibiting remarkable thermodynamic and kinetic stability [1]. While G-quadruplexes form readily in vitro from single nucleic acid strands, their assembly and stabilization in vivo, where they may exist in equilibrium with a different type of structure (e.g., double-stranded DNA), has been suggested to require the function of protein chaperons [2]. 

**Figure 1:**
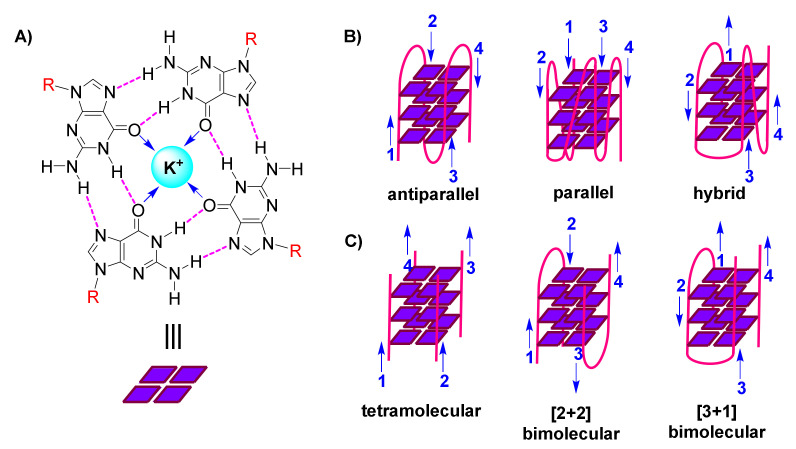
(**A**). Representation of a guanine(G)-quartet, highlighting the network of Hoogsteen hydrogen bonds (magenta), monovalent cation (cyan), dipole-cation interactions (blue arrows), and sites of connection to the sugar-phosphodiester backbone (R, red) (**B**). Cartoon representations of diverse unimolecular/intramolecular G-quadruplexes, with blue arrows indicating direction of each strand (numbered) (**C**). Cartoon representations of diverse intermolecular G-quadruplexes, with blue arrows indicating direction of each strand (numbered).

The following organization is characteristic of a G-quadruplex assembly: Guanines from the participating sequence(s), in sets of four, are oriented in square planar quartets, driven by a network of Hoogsteen hydrogen bonds (Figure 1A); G-quartet stability is further enhanced by coordination of (monovalent) cations to guanine carbonyls (Figure 1A); and G-quartets accumulate atop each other due to π-π stacking, while interconnected by the sugar-phosphodiester backbone (Figure 1B,C) [3,4,5]. 

G-quadruplexes are polymorphic entities, as revealed by 3D structural studies, with their family comprising both unimolecular/intramolecular (Figure 1B) and intermolecular (Figure 1C) structures. These exhibit diversity in the lengths, sequences, folds and orientations of the loops that interconnect the participating strands, leading to classification of G-quadruplexes as parallel, antiparallel or hybrid (Figure 1B) [6,7,8].

Since the early days of this field, in attempting to answer the question whether G-quadruplexes are biologically relevant, algorithms have been devised and applied by various research teams, in order to predict possibility of occurrence of G-quadruplexes in the human and other genomes [9,10,11,12]. Genome-wide analyses have indicated a frequent occurrence of G-quadruplex-forming sequences in functional genomic regions, suggesting G-quadruplex association with telomere maintenance, replication, transcription and translation which, in turn, has led to suggestions of G-quadruplex-mediated regulatory mechanisms for these processes. The roles of G-quadruplexes in these processes are understood in much detail today [13]. 

Many of the >370,000 predicted G-quadruplex-forming sequences in humans [9,10] are traced in promoter regions of genes, close to transcription start sites [12]. Despite the fact that these predominantly exist in vivo in the form of double-stranded helices, their transient conversion to single-stranded is believed possible, in the course of replication, transcription and recombination. It can be achieved with the assistance of negative DNA supercoiling and conditions of molecular crowding, caused by protein binding, which favor folding into G-quadruplexes [14]. Moreover, the presence of tandem G-rich repeats in the human telomere [15,16], which is naturally single-stranded, energetically favors formation of multiple G-quadruplexes. On the other hand, RNAs containing G-quadruplex-forming motifs in their 5’-untranslated regions (5’-UTRs), estimated to be around 3000 in humans [17], are also single-stranded and readily fold into stable G-quadruplex structures. 

Most G-quadruplex-related studies have been conducted ex vivo. However, accumulating experimental evidence is now providing proof of the in vivo occurrence of G-quadruplexes. An early study employing high-specificity antibodies against telomeric G-quadruplexes, raised by ribosome display, has achieved targeting of intermolecular antiparallel G-quadruplexes in the ciliate model organism *Stylonychia* [18]. More recent studies involving highly specific antibodies, have achieved visualization of G-quadruplexes in living human cancer cells [19,20] and tissues [21]. Also, over the last few years, there has been significant progress in the development of G-quadruplex-specific, small-molecule-based fluorescent probes and theranostics [22,23,24,25,26,27], which now find application as bioimaging agents to trace G-quadruplexes in a cellular context and expand our understanding on their functional roles in physiological processes, including those with consequences for cancer research.

The presence of G-quadruplex-forming motifs in key genomic DNA and RNA sequences, uniquely places them in position to regulate several cellular pathways. Importantly, many of these pathways are directly associated to well-established hallmarks of cancer [28]. Indicatively, G-quadruplexes have been correlated to chromosomal homeostasis, genome maintenance and integrity, apoptosis and survival, proto-oncogene and cancer protein expression and post-translational modifications [13]. G-quadruplex-forming sequences are often found amplified in certain cancers [29,30]. The realization of a strong link between G-quadruplexes and unprecedented anticancer mechanisms of action has leveraged G-quadruplex structures to therapeutic target status in oncology [31,32,33]. The physiological relevance and significance of G-quadruplexes in the context of cancer have been widely reviewed [34,35,36]. 

The putative roles of G-quadruplexes in prevention of cancer pathogenesis have been, for years, a major inspiration and drive for research efforts by many teams, with implications from a pharmacological perspective, for the design of small-molecule ligands targeting G-quadruplexes and aiming to induce G-quadruplex-mediated anticancer effects. A vast number of scaffolds have been proposed and new compounds designed and synthesized to address the task at hand, namely the binding (with high affinity and selectivity) and stabilization of G-quadruplexes in nucleic acid sequences of cancer relevance [36,37,38,39,40,41,42]. Cellular responses upon treatment of cells with G-quadruplex-targeting ligands have been correlated with the perceived function of these G-quadruplexes. In parallel, several methodologies for ascertaining the anticancer potential of G-quadruplex-stabilizing ligands have been described [43]. 

The present review focuses on advancements of the last few years in the development of promising G-quadruplex-targeted ligands, exhibiting interest from an anticancer drug development perspective. The review aims to discuss the most common pathways via which G-quadruplexes may exert their anticancer activities, as well as promising case studies of lead compounds for which there is now cellular, in vivo or clinical data available, and which exhibit drug-like features. Finally, the review considers limitations in the development of such lead compounds, that have hampered in the past their progression to clinical trials, and provides a future outlook on how these may be circumvented, to allow harnessing the anticancer potential of G-quadruplexes.

The scope of this review encompasses exclusively small-molecule-type ligands for G-quadruplex targeting. While we will not discuss cases of aptamers against G-quadruplexes herein, it should be noted that aptamers compose a separate class of G-quadruplex modulators in their own right, whose remarkable selectivity against specific G-quadruplexes promotes anticancer effects [44]. Apart from serving as targets for aptamers, G-quadruplexes themselves may successfully play the role of aptamer against other types of biomolecular targets, such as cancer-implicated proteins, to provide valuable therapeutic possibilities, by eliciting potent antiproliferative effects in various cancer cell lines [45,46,47]. 

## 2. G-Quadruplex-Mediated Anticancer Mechanisms 

### 2.1. Interference with Chromosomal Homeostasis & Telomerase-Mediated Telomere Elongation

The telomere is a region of repetitive nucleotide sequences at chromosomal ends which, via complexation with various nucleoproteins, folds into higher-order secondary structures, that play the role of a ‘cap’, protecting the chromosome from deterioration or fusion with other chromosomes [48]. The type of ‘cap’ secondary structure and the participating proteins exhibit variability between different species [2,49]. The existence of an intact ‘cap’ also prevents improper activation of DNA damage-response pathways [50].

G-quadruplexes occur in high concentrations in telomeres [19,51], due to the high guanine content of the telomere tandem sequence (TTAGGG in vertebrates) and are, in fact, capable of protecting genome integrity in cases where normal telomeric ‘caps’ are compromised [52]. In vitro studies have shown telomeric G-quadruplexes to interact with human proteins TRF2, EWS and FUS, which can co-bind the long non-coding RNA TERRA [53,54,55]. The simultaneous binding of telomeric and TERRA G-quadruplexes causes recruitment of histone methyltransferases by FUS, thus providing an association with telomere heterochromatin maintenance [54].

Stabilization of G-quadruplexes in the telomere during DNA replication could generate problems. Loss of telomeric G-quadruplex-interacting proteins, such as the CST cluster [56] and RTEL1 helicase [57], results in telomere shortening and fragility, and affords altered rates of replication [58]. The addition of G-quadruplex-stabilizing ligands was shown to exacerbate this situation [56]. 

**Figure 2:**
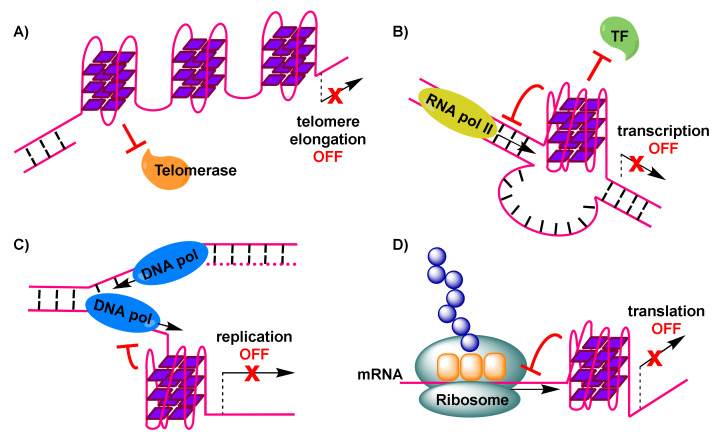
Formation of G-quadruplexes impacting physiological processes, with anticancer consequences: (**A**) G-quadruplexes in the telomere impose hindrance to telomerase, preventing elongation of the telomere and triggering DNA damage response signals. (**B**) G-quadruplex in oncogene-promoter region dislocates transcription factors and down-regulates RNA polymerase-mediated transcription of (onco)genes. (**C**) G-quadruplex in DNA undergoing replication stalls replication fork progression and leads to replicative stress, resulting in double strand breakpoints. (**D**) G-quadruplex in mRNA interferes with translation and formation of cancer proteins.

Importantly, telomerase, a reverse transcriptase that is over-expressed in about 85% of cancer cells [59], stem cells and germline cells, is responsible for providing genomic stability by elongating the protruding 3’ single-stranded G-rich overhang at the ends of telomeres. For this extension to be permitted, base pairing needs to take place between the G-rich overhang and a RNA template carried by telomerase to encode the telomeric repeat sequence [60]. Elongation, which counteracts telomere shortening, may be inhibited by the formation of G-quadruplexes in telomeric sequences [61]. This is a result of hindered access of telomerase to the telomere sequence, caused by formation of antiparallel intramolecular G-quadruplexes (Figure 2A). However, alternative intermolecular parallel G-quadruplexes may also form, which can be partially resolved by telomerase in vitro, allowing the extension to proceed [62]. Evidence from *Saccharomyces cerevisiae* indicates a co-localization of parallel G-quadruplexes in the telomere with telomerase [62,63]. On the other hand, the POT1-TPP1 protein complex, responsible for recruitment of telomerase to the telomeres, is capable of destabilizing G-quadruplexes [64,65]. Recent evidence shows the importance of G-quadruplex formation in a POT1-TPP1 mediated DNA synthesis [66]. Finally, telomerase activity may be affected by the 5’ end unfolding of its RNA component, caused by a small molecule [67]. 

A vast number of ligands to stabilize telomeric G-quadruplexes in cancer cells have been described, despite the natural role of G-quadruplexes in telomerase-mediated telomere elongation not being fully elucidated. A resulting inhibition of telomerase activity upon addition of such ligands has been reported [68], while several ligands are able to displace members of the telomere protection complex shelterin, resulting in telomere damage and cell death [59,69]. While an alternative path of telomere elongation may be promoted upon G-quadruplex-imposed replication stress in certain cancer cells [70], presence of a G-quadruplex-stabilizing ligand may still result in cell death [71].

### 2.2. Transcriptional Regulation of Proto-Oncogene Promoters

The early detection, by means of applying computational predictive algorithms [12], of G-quadruplex-forming motifs in the promoter regions of several known proto-oncogenes [72], has indicated that G-quadruplexes are over-represented in these regions and may, in fact, possess regulatory roles with regard to the expression levels of oncogenes. Additional efforts have been successful in mapping G-quadruplex structures in chromatin to regulatory regions found adjacent to the transcription start sites of several of these genes in humans [30,73]. 

A number of in vitro studies applying small-molecule G-quadruplex-targeted ligands as agents inducing stabilization of G-quadruplexes in proto-oncogene promoter regions have demonstrated an ensuing reduction in oncogene transcription levels. Examples include transcriptional regulation of *MYC, KRAS, KIT, BCL2 and VEGF* [72,74,75,76,77]. However, explicit evidence of a link between G-quadruplexes and transcriptional control, coming from cellular studies, remains quite limited [78].

Indirect evidence of G-quadruplex impact on transcription of oncogenes is provided by the fact that certain transcription factors recognize G-quadruplex structures in vitro. Examples include recombinant nucleolin recognizing *MYC* [79], CNBP recognizing *MYC* [80] and SP1 recognizing *ΚΙΤ* [81]. This has led to the hypothesis that G-quadruplex-mediated mechanisms may be employed by nature for transcriptional regulation purposes. 

To explain reduced expression levels of oncogenes, it has been suggested that G-quadruplex formation may impair initiation of transcription by preventing binding of RNA polymerase II and transcriptional machinery to the promoter transcription start site (Figure 2B) [74].

The formation of a G-quadruplex in the human telomerase reverse transcriptase gene (*hTERT*) has also been suggested to prevent binding of the gene repressor CCCTC binding factor, leading, in this case, to elevation of plasmid-encoded *hTERT* transcription [82].

### 2.3. Ribosomal DNA (rDNA) Transcription Inhibition

Ribosomal DNA (rDNA) is a GC-rich DNA sequence located in the nucleolus of cells, which encodes for ribosomal RNA. It contains more than 400 copies of the rRNA genes, organized in tandem arrays.

Ribosome biogenesis is under the control of multiple cellular signaling pathways, converging on the RNA polymerase I complex. RNA polymerase I is responsible for the transcription of rRNA genes and production of pre-rRNAs which, after maturation, will provide the main components for construction of the ribosome.

In cancer cells, proto-oncogene ‘gain-of-function’ and tumor-suppressor ‘loss-of-function’ mutations operate, leading to deregulated cellular signaling pathways, which in turn results in excessive ribosome biogenesis, required to support the rapid cell proliferation in tumors [83,84,85,86]. Given that the synthesis of rRNA by RNA polymerase I is considered the rate-limiting step in ribosome biogenesis [87], the interaction of rDNA with the RNA polymerase I protein complex could be a locus for anticancer intervention. Disruption of this interaction leads to arrest of ribosome biogenesis.

G-quadruplexes are believed to have a role in rDNA transcription. Specifically, G-quadruplexes may form transiently in the non-template strand in the course of rDNA transcription, and their occurrence prevents renaturation of the template DNA, assisting toward a dense arrangement of RNA polymerase I molecules on rRNA genes [88]. The formation of G-quadruplexes appears to be associated with their nanomolar-affinity interaction with nucleolin [89], an abundant nucleolar protein whose presence is essential for the progression of rDNA transcription [90]. Therefore, the disruption of G-quadruplex-nucleolin association, by means of interference with small-molecule ligands, is a way of inhibiting RNA polymerase I-mediated rDNA transcription, leading up to ribosome biogenesis suppression and eventually apoptosis of cancer cells (for examples, see Section 3.3).

### 2.4. Induction of Replication Stress Causing Genome Instability

Formation of G-quadruplexes in DNA sites during the transient opening of the double helix in the course of replication, has been implicated in increasing replication stress [91]. This is the result of obstruction caused to the progression of the replication forks (Figure 2C), leading to replication-fork collapse [92,93] and eventually the generation of double-strand breakpoints that cause genome instability and pose a threat to cell viability. 

Via use of computational analyses of cancer databases, G-quadruplex formation has been associated with breakpoints in many cases in cancer cells, relevant to somatic copy-number alterations [94]. Stable G-quadruplexes were also found to be enriched in sites of somatic mutations, suggesting they may have roles as important determinants of mutagenesis [95]. G-quadruplex sequencing in the human genome has also revealed correlations of G-quadruplexes with gene amplifications, observed in cancer cells [29,30].

Evidence of genome instability due to G-quadruplex formation in the course of replication comes from elaborate studies in the model organisms *Caenorhabditis elegans*, *Saccharomyces cerevisiae* and *Xenopus laevis*, where the knock-out of a rescue system, namely helicases with the ability to resolve G-quadruplexes (such as DOG1, FANCJ and PIF1), renders the system prone to occurrence of DNA breakpoints [96,97,98,99,100,101]. These findings highlight the importance of helicases in cellular rescue mechanisms, as well as the relation between potential helicase ‘loss-of-function’ and genome instability.

### 2.5. Interference with Translation of Messenger RNA (mRNA) to Cancer Proteins

The bioinformatics discovery that G-quadruplex-forming motifs are prevalent in 5’-UTRs of RNAs [15], confirmed by spectroscopic studies on these sequences, has rendered such mRNA transcripts that encode for proteins with functional roles in cancer, attractive targets. The 5’-UTRs of mRNAs are located adjacent to translation initiation sites. Therefore, the formation of G-quadruplexes in 5’-UTRs of mRNA sequences (Figure 2D) may result in interference with mRNA translation [102] (e.g., potential formation of the ribosome at alternative, upstream start codons, thus preventing translation of the main open reading frame [103]), eventually depriving cancer cells of valuable proteins. An early prototype example, of interest to anticancer research, is the 5’-UTR of NRAS mRNA, where emergence of a G-quadruplex has been correlated with about 80% repression in protein levels in vitro, based on a luciferase reporter assay [17]. Many subsequent efforts, including studies in live cells, have identified additional G-quadruplex-forming sites in 5’-UTRs of the same and other mRNAs, which can be manipulated, by means of stabilization by appropriate small-molecule ligands, to achieve similar impact on translation (for recent examples, see Section 3.4). 

G-rich sequences within mRNA coding regions are also encountered, however, at lower abundance compared to 5’-UTRs [104]. Upon G-quadruplex formation, they exhibit ability to stall translation, 6-7 nucleotides before the G-quadruplex [105].

The above findings, in addition to the identification of helicases capable of unwinding RNA G-quadruplexes [103], supports the notion that RNA G-quadruplexes may serve as a natural mechanism of regulating the expression levels of specific genes on a post-transcriptional level. 

Small-molecule-based tools that offer the ability to modulate the stability of G-quadruplexes of this type, in a dose- and time-dependent manner, can be pharmacologically useful, especially given the single-stranded nature of mRNAs, which makes them more susceptible to modulation compared to dsDNAs.

## 3. Case Studies of Selected G-Quadruplex-Stabilizing Ligands with Available Cellular or In Vivo Evaluation Results

### 3.1. Ligands Acting on the Human Telomeric Sequence

**Figure 3:**
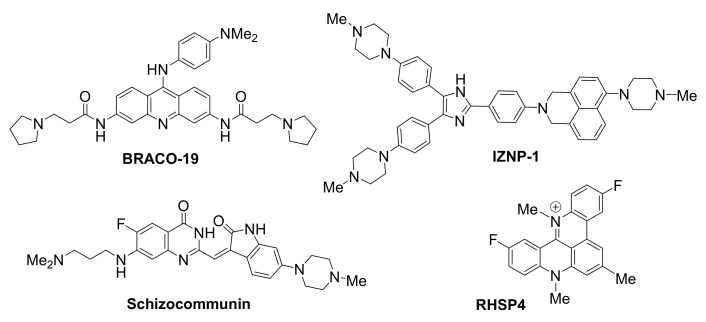
Structures of telomeric G-quadruplex-targeted ligands.

The telomere has been an early target for small-molecule ligands (Figure 3), aiming to arrest activity of telomerase, the enzyme responsible for telomere elongation, a pre-requisite for cancer cell ‘immortalization’. The stabilization of a G-quadruplex in the human telomere induces telomere shortening and apoptosis or senescence of tumor cells [106], consistent with telomerase function impairment [107].

BRACO19, an aminoacridine, was shown to be one of the first potent and selective stabilizers of G-quadruplexes in telomeres, resulting in telomerase inhibition [108]. Treatment of glioblastoma cells with BRACO19 caused uncapping of the chromosomes, exposing them to damage, thus triggering a DNA damage response. Specifically, disassembly of the T-loop was observed, accompanied by displacement of TRF2 and POT1 (two components of the shelterin complex), ultimately leading to p53- and p21-mediated cell cycle arrest, short-term apoptosis and senescence.

Natural sources have also provided significant scaffolds as a basis for the development of G-quadruplex ligands. Schizocommunin, an alkaloid from a fungal source, has been derivatized to provide efficient telomeric G-quadruplex stabilizing ligands [109]. G-quadruplex occurrence in the telomeres upon exposure of cancer cells to a schizocommunin analogue has been demonstrated via use of a BG4 antibody in the nucleus. A DNA damage response was triggered and proteins TRF2 and POT1 were displaced, causing telomere uncapping and leading to production of anaphase bridges. In a cervical squamous cancer xenograft mouse model, the same derivative inhibited tumor growth, while maintaining a low toxicity profile. 

RHSP4, a synthetic, cationic, planar telomere G-quadruplex-targeting ligand, exhibited strong growth inhibition activity in various in vivo tumor models, including a recent application as a radio-sensitizing agent in glioblastoma multiforme xenograft model [110]. Ionizing radiation (IR) was employed to activate the agent, leading to effective tumor growth inhibition for up to 65 days. Effects of this RHSP4-IR combined treatment on the telomere were detected in the initial stages of treatment, thus enhancing cellular sensitivity to the small molecule. While RHSP4-IR exposure did not produce a similar telomere damage in glioma stem-like cells, treatment with RHSP4 alone was able to arrest growth in these cells, believed to be due to induction of replicative stress by means of RAD51 and CHK1 depletion.

Compound IZNP-1 has been recently reported to specifically target and stabilize multimeric G-quadruplexes formed in the 3′-end microsatellite repetitive sequence of the telomere [111]. The compound is proposed to bind, with high affinity and via intercalative mode, in the pocket between 2 sequential G-quadruplexes. Its binding is reported to promote cell cycle arrest, apoptosis and senescence in Siha cancer cells, due to telomeric DNA damage and telomere dysfunction caused by the interaction of the molecule with G-quadruplexes. Selectivity is demonstrated by the fact that treatment with the molecule does not have any observed effect on the transcriptional levels of many known oncogenes with a G-forming element in their promoter.

### 3.2. Ligands Acting on Oncogene Promoters

A functional role for G-quadruplexes in transcriptional regulation, selected for by evolution, has been proposed [11,112], due to the frequent occurrence of G-quadruplex-forming motifs in promoter regions upstream of the transcription start sites of many human genes [12,113]. A plausible anticancer therapeutic approach exploiting G-quadruplexes involves suppression of oncogene expression by inducing a G-quadruplex structure in the promoter regions upstream of these genes. Selective ligands have been reported for a number of proto-oncogene promoters. 

#### 3.2.1. MYC

**Figure 4:**
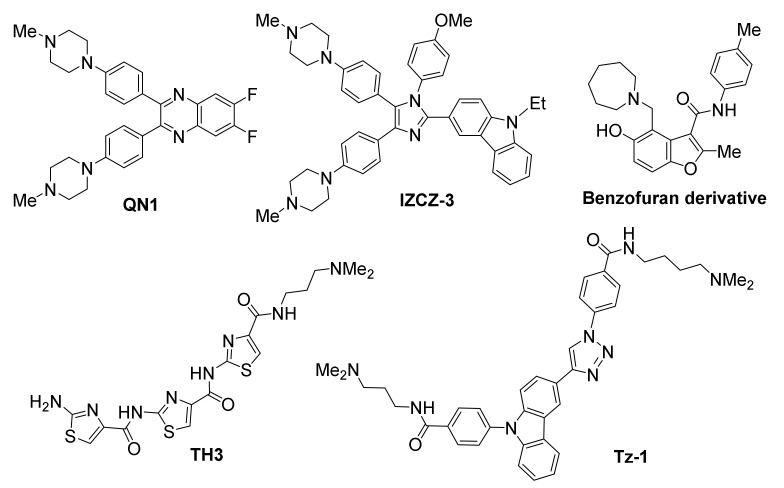
Structures of *MYC* promoter G-quadruplex-targeted ligands.

*MYC* overexpression is encountered in about 80% of all solid tumors, including gastrointestinal, breast and ovarian, as well as in non-Hodgkin’s lymphoma [114,115,116]. The fact that the protein product of *MYC*, a transcription factor, is considered non-druggable [117], renders intervention on the DNA level an ideal approach for anticancer effect. The *MYC* proto-oncogene, first found to contain a possible G-quadruplex-formation site in the nuclease hypersensitive element III_1_ (NHE III_1_) of its promoter [75], has recently been targeted by a series of promising ligands (Figure 4). 

The quinoxaline QN1 has been demonstrated to efficiently suppress tumor growth in a triple-negative breast mouse model, believed to be a result of its selective action on *MYC* promoter [118]. Evidence provided, showed selective downregulation of *MYC* expression, while not affecting other G-quadruplex-forming proto-oncogene promoters, such as *BCL2*, *KIT*, *VEGF* and *HRAS*. The same study revealed downregulation of cyclin D1, an effector downstream of *MYC*, suggesting a *MYC*-specific mechanism of action for QN1. Inhibition of *MYC* transcription was found to be G-quadruplex-mediated, leading up to cell cycle arrest and apoptosis.

A “four-leaf clover-like” imidazole/carbazole-based compound named IZCZ-3, with partial structural similarity to QN1, also exhibited a promising profile against *MYC* [119]. While it demonstrated a high affinity for the *MYC* promoter-derived G-quadruplex, it also showed considerable inhibitory potential against cancer cell lines, by inducing cell cycle arrest at G0/G1 and apoptosis. This behavior was correlated to downregulation of *MYC*, cyclin D1 and CDK6, as well as upregulation of a number of apoptosis regulators, while not affecting β-actin. The mechanism of action for IZCZ-3 was found to be *MYC*-specific, without targeting other G-quadruplex-forming promoters, such as *KRAS*, *KIT*, *VEGF*, *BCL2*, *HRAS*, *RET* and *PDGFA*. Treatment of mice bearing human cervical squamous cancer xenografts with IZCZ-3 exhibited a similar *MYC*-mediated antitumoral effect, comparable to doxorubicin’s. Importantly, cytotoxicity of this compound against human normal cells or primary mouse cells was low, suggesting reduced side effects.

The thiazole-based peptide TH3 has also shown promise, binding *MYC* with high affinity while exhibiting preference for *MYC* over *BCL2* and *KIT* [120]. NMR studies have suggested a end-stacking rather than groove binding mechanism of action, with the ligand also interacting with a AT-rich capping structure at both G-quadruplex ends that is unique to *MYC*. Cellular studies have shown good uptake and nuclear localization of the compound, resulting in cell cycle arrest and induction of apoptosis. In this case as well, the observed effects were shown to be *MYC*-specific, while no alteration of *BCL2* or non-cancer *GAPDH* and 18S rRNA control genes were observed. 

A benzofuran derivative was identified out of a high-throughput microarray screening effort evaluating 20,000 compounds, which employed a fluorescently-labelled *MYC* promoter DNA to isolate *MYC*-selective binders [121]. Its ability to bind the *MYC* promoter and downregulate its transcription was demonstrated via SPR-binding assay and PCR-stop assay, respectively. The compound was found to reduce cell viability in myeloma cell lines, while exhibiting negligible effects on cells harboring a *MYC* translocation that depleted the G-quadruplex-forming motif, as well as in normal blood mononucleocytes. Moreover, the compound demonstrated excellent selectivity, since it did not alter transcriptional levels of other oncogenes with G-quadruplex-forming elements in their promoters (*BCL2*, *KRAS*, *HIFA*, *VEGF*, *Rb1*). 

A carbazole/triazole hybrid (Tz-1) was also identified as a *MYC*-interacting ligand through a building block-selecting, target DNA-guided screening approach [122]. Tz-1 was shown to cause excellent suppression of the oncogene at low µM concentrations in HCT116 colorectal carcinoma cell cultures. A *MYC* G-quadruplex-mediated mechanism of action was demonstrated in CA46 Burkitt’s lymphoma cells.

#### 3.2.2. KIT

**Figure 5:**
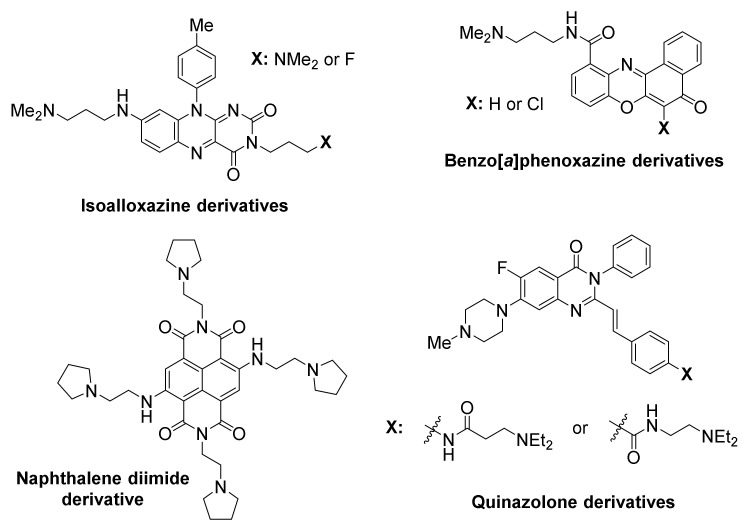
Structures of *KIT* promoter G-quadruplex-targeted ligands.

The *KIT* proto-oncogene encodes a receptor tyrosine kinase that receives extracellular signals and is involved in proliferation, differentiation and survival in hemopoietic cells [123,124,125]. The protein product of *KIT* is a clinically validated target for gastrointestinal stromal tumors [126]. One way of suppressing *KIT* activity is the stabilization of G-quadruplexes in appropriate sites of its promoter region, via use of *KIT*-selective small-molecule ligands (Figure 5). Two such sites have been identified [127,128].

Members of an isoalloxazine family of ligands demonstrated a binding preference to *KIT* G-quadruplexes over a telomeric G-quadruplex, and inhibited *KIT* oncogene expression in two cancer cell lines [77].

A naphthalene diimide derivative also showed stabilization of *KIT* G-quadruplexes accompanied by reduction of encoded protein levels, leading to an arrest of cell growth in patient-derived GIST tumor cells [129].

Additional ligand classes were also identified in cell-based screening efforts as *KIT* G-quadruplex-stabilizing ligands, with ability to suppress transcription and cause cancer cell growth inhibition, including benzo[*a*]phenoxazines [130] and quinazolones [131]. 

#### 3.2.3. KRAS

**Figure 6:**
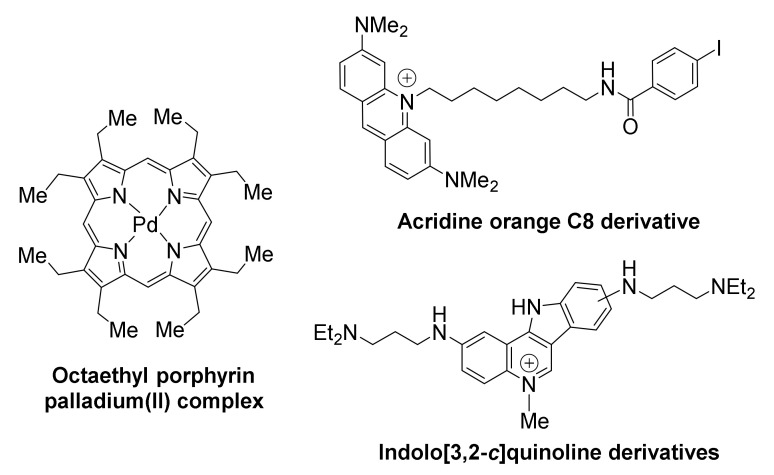
Structures of *KRAS* promoter G-quadruplex-targeted ligands.

The *KRAS* oncogene promoter has arisen as another potential target for compounds of antitumoral potential (Figure 6). *KRAS* mutations are major oncogenic driver mutations in many cancers and considered of critical importance in acquisition of drug resistance by cancer cells [132,133].

Compounds belonging to the family of porphyrins have been developed as anticancer therapies for pancreatic cancer. Examples include the tetra- and octa-ethyl porphyrin palladium complexes, found to stabilize the G-quadruplex in *KRAS* promoter and suppress *KRAS* transcription in both PANC-1 and Mia PaCa 2 pancreatic cancer cells, while upregulating apoptosis response elements p53 and Bax, thus triggering apoptosis [134]. It is possible that the effects of these compounds are not *KRAS*-specific, as other G-quadruplex-forming promoters (i.e., *BCL2*) appear to be also downregulated. Both porphyrins inhibit metastasis, by arresting the epithelial to mesenchymal transition, characteristic of pancreatic cancer. 

The acridine orange C8 derivative is another example of a high-affinity ligand for *KRAS* [135]. It exhibits high inhibitory activity against HeLa cervical cancer cells, with IC50 value being two orders of magnitude lower than that of the anticancer drug 5-fluorouracil. Downregulation of *KRAS* transcriptional levels is selective, with β-actin not being affected. The compound exhibits efficient uptake by cells and localization at the nucleoli of HeLa cells. 

Members of a family of indolo[3,2-*c*]quinolines that exhibited triple-cationic features, showed binding preference for *KRAS* G-quadruplex over telomeric G-quadruplex and dsDNA, were effective in significantly downregulating *KRAS* expression, and inhibited mutant *KRAS* expression in HCT116 and SW620 cancer cells [136].

#### 3.2.4. VEGF

**Figure 7:**
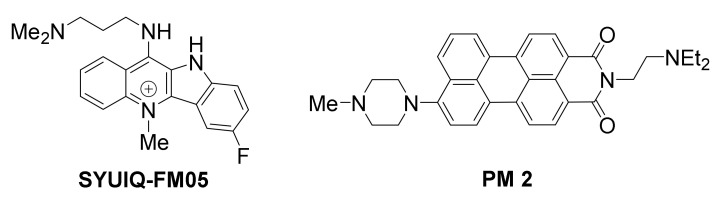
Structures of *VEGF* promoter G-quadruplex-targeted ligands.

*VEGF* has arisen as a compelling target in anti-cancer research, since it plays a role in neovascularization of tumors, and is found to be over-expressed in many cancer cells [137,138]. It exhibits five arrays of G-tracts upstream of its transcription start site, enabling formation of G-quadruplexes [139,140], that can be targeted by small molecules (Figure 7).

A quindoline-based ligand, SYUIQ-FM05, was shown to engage in strong interaction with *VEGF* G-quadruplexes, exhibiting considerable antiangiogenic and antitumor activity [141].

Asymmetric perylene monoimide derivative PM 2 was shown to induce and stabilize a *VEGF* G-quadruplex in vitro [142], in presence of competitive duplex DNA. PM 2 was evaluated in living A549 (lung) cancer cells, and found to suppress *VEGF* gene expression, as well as *VEGF* protein expression, in a dose-dependent manner. The levels of *VEGF* gene downregulation were directly comparable with levels of G-quadruplex induction in the duplex/G-quadruplex competition assay, suggesting that the gene suppression is G-quadruplex-mediated. The expression of control genes *GAPDH*, *Max* and *Cox-2*, with no potential for G-quadruplex formation in their promoters, was not affected, however the expression of oncogenes *MYC* and *BCL2* with G-quadruplex-forming promoters was reduced, suggesting broad selectivity of this ligand for G-quadruplexes. Similar results were obtained in cancer cell lines MCF-7 (breast), HCT15 (colon) and HeLa (cervical).

#### 3.2.5. BCL2

**Figure 8:**
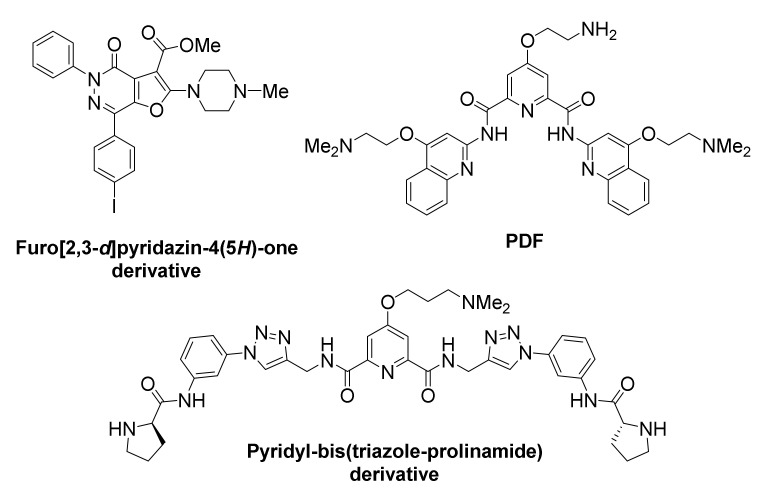
Structures of *BCL2* promoter G-quadruplex-targeted ligands.

*BCL2* oncogene overexpression is associated with aberrant carcinoma growth, particularly in association to solid tumors [143]. It is also considered a hallmark of chemoresistance [144,145]. Multiple sites have been identified in *BCL2*, capable of folding into G-quadruplexes [146,147,148]. Several reports on newly-developed small-molecule ligands involve action on *BCL2* G-quadruplexes (Figure 8).

A furo[2,3-*d*]pyridazine-4(5*H*)-one derivative was identified, featuring binding preference for *BCL2* G-quadruplexes over *KIT*, *MYC*, telomere G-quadruplexes and dsDNA [149]. This compound successfully suppressed *BCL2* expression and led to high cytotoxicity in Jurkat (human acute T cell leukemia) cell lines.

A new pyridostatin analog, referred to as PDF, exhibited high selectivity and stabilizing ability toward the *BCL2* promoter G-quadruplex vs. dsDNAs [150]. However, discriminatory ability for diverse G-quadruplexes was not evaluated. Treatment of human laryngeal squamous carcinoma (Hep-2) cells with the compound led to significant suppression of *BCL2* transcription and apoptosis.

A symmetric pyridyl-bis(triazole-prolinamide) ligand has been developed, which exhibits not only high affinity for *BCL2* (and for *MYC*) promoter G-quadruplex, but also remarkable selectivity in promoting their in vitro stabilization, compared to other G-quadruplexes, such as *KIT1*, *KIT2*, *KRAS*, *VEGF* and telomeric, as well as dsDNA [151]. A combination of real-time quantitative reverse transcription, Western blot, dual luciferase and small interfering RNA knockdown assays suggested that this ligand simultaneously inhibits the expression of *BCL2* and *MYC* through their promoter G-quadruplexes, thus inducing synthetic lethality. Significant growth inhibition was observed in MCF-7 cancer cells that overexpressed both *BCL2* and *MYC* genes, and less so in cells that overexpress either of the two. Treatment of cells with the ligand induces S-phase cell cycle arrest, DNA damage response and apoptosis.

#### 3.2.6. hTERT

**Figure 9:**
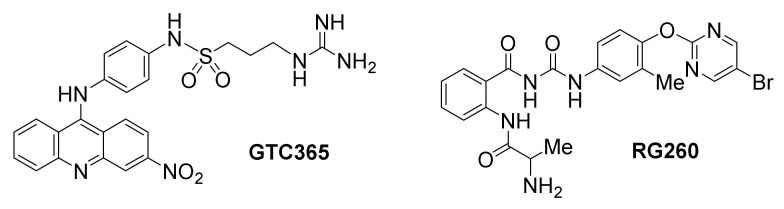
Structures of *hTERT* promoter G-quadruplex-targeted ligands.

The *hTERT* gene encodes the catalytic subunit of telomerase, and the fact that it is overexpressed in about 85% of all cancers while it is silent in most normal cells, has rendered it a compelling target for anticancer research [152]. Elevated levels of *hTERT* in cancer patients are associated with low survival rates [153]. Multiple sites for G-quadruplex formation exist in the promoter region upstream of the *hTERT* gene. It has been proposed that the formation of tandemly aligned G-quadruplexes serves as a mechanism for maintaining normal transcriptional levels of *hTERT* gene, while occurrence of mutations has been associated with the observed elevated expression levels in cancer [154,155]. Two recent examples of *hTERT*-targeted ligands are discussed herein (Figure 9).

The small molecule GTC365 has been developed to bind high-order *hTERT* promoter G-quadruplexes [155]. It is designed to use a dual targeting motif against both the G-quadruplex core and a mis-matched duplex stem loop found in the G-quadruplex structure. The occurrence of mutations in the mismatched duplex stem loop are thought to cause misfolding alterations of the G-quadruplexes, resulting in *hTERT* overexpression, likely by inhibiting binding of transcriptional negative regulators. However, binding of GTC365 is proposed to counterbalance the folding alterations, establishing a similar pattern as in the wild type *hTERT* G-quadruplex. The antitumor activity of this compound was evaluated in MCF-7 breast cancer cells, resulting in apoptosis and senescence.

Another promising ligand by the same team, which lacks the acridine moiety of GTC365 but maintains an analogous chaperon-like ability to modulate *hTERT* G-quadruplex folding, is benzoylphenylurea RG260 [156]. Specifically, this ligand is proposed to target the *hTERT* G-quadruplex hairpin stem loop folding rather than directly interacting with G-quartets, resulting in downregulation of *hTERT* transcription. RG260 induced apoptotic cell death in several human prostate cancer cell lines, but not in mouse prostate epithelial cancer cells, which lack the unique stem loop-containing G-quadruplex. A number of bioavailability-optimized analogs of RG260 were developed in the same study, one of which (RG1603) exhibited significant growth inhibition of established human prostate tumors in mouse xenograft models.

### 3.3. Ligands Acting on Ribosomal DNA

**Figure 10:**
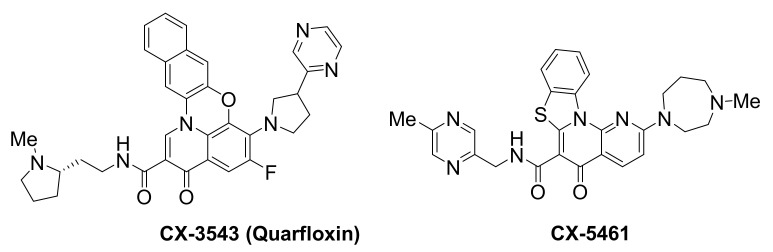
Structures of rDNA G-quadruplex-targeted ligands that have progressed to clinical trials.

The transcription of ribosomal DNA (rDNA) is an important target for anticancer research, since it is the rate-limiting step for rRNA biogenesis in cancer cells. Notably, quinolone-based small molecules found to target this system (Figure 10) are the first examples of G-quadruplex-binding ligands that have progressed to clinical trials.

Small-molecule CX-3543, also known as quarfloxin, was reported to target and selectively disrupt nucleolin/rDNA G-quadruplex complexes in the nucleolus, leading to inhibition of RNA polymerase 1-mediated transcription and induction of apoptosis in cancer cells [157]. CX-3543 accumulation to the nucleolus was accompanied by nucleolin displacement from the nucleoli to the nucleoplasm, prior to rRNA synthesis inhibition, in A549 lung carcinoma cells, as well as in p-53 null human osteosarcoma cells, Saos-2. This indicated a direct effect of CX-3543 rather than a p53-mediated stress response. Selectivity for nucleolin was demonstrated by showing that CX-3543 had no effect on the binding of other nucleolar proteins, such as fibrillarin. Selectivity toward inhibition of rRNA synthesis vs. DNA synthesis was also demonstrated. The compound had no effect on RNA polymerase II-mediated transcription of oncogenes *BCL2*, *MYC*, *MYB* and *KRAS*, and did not cause any telomere-related dysfunctions. Broad antiproliferative activity in vitro, and antitumor activity in vivo, in murine xenograft models of multiple human cancers, was shown. This example represents a novel approach for selectively disrupting cancer cell proliferation, rendering CX-3543 the first G-quadruplex-interacting agent to enter clinical trials and reach phase II, undergoing evaluation against carcinoid/neuroendocrine tumors (NCT00780663).

CX-5461 is a structurally-related quinolone [158], sharing the same biological target, namely the rDNA transcription, which is greatly upregulated in cancer cells in order to meet their elevated demand for protein synthesis. CX-5461 binds to rDNA G-quadruplexes and inhibits rDNA transcription, by reducing the binding affinity of the SL1 pre-initiation complex and RNA polymerase I complex toward rDNA promoters. This conveys p53-mediated anti-tumor activity in hematopoietic malignancies [159,160]. In addition to rDNA G-quadruplexes, additional targets are reported for this ligand, including activation of ATM/ATR [161] and rapamycin-associated signalling [162]. CX-5461 is now in phase I clinical trials for patients with BRCA1/2-deficient tumours (NCT02719977). BRCA2 deficiencies have been correlated to compromised homologous recombination-mediated DNA damage repair, leading to error-prone repair and ultimately genomic instability [163]. This ligand is expected to offer a novel therapeutic approach against cancers with somatic inactivation of HR pathway genes. CX-5461 exhibits specific toxicity against BRCA deficiencies in both cancer cells and polyclonal patient-derived xenograft models, including tumors resistant to PARP inhibition [158]. Treatment of cells with this ligand, blocks replication forks and induces breaks or gaps to ssDNA. This can be attributed to stabilization of G-quadruplexes, which has been associated with increased tendency for DNA damages [164]. Because BRCA pathway is needed to repair such DNA damages, failure to do so results in lethality.

### 3.4. Ligands Acting on Messenger RNA

**Figure 11:**
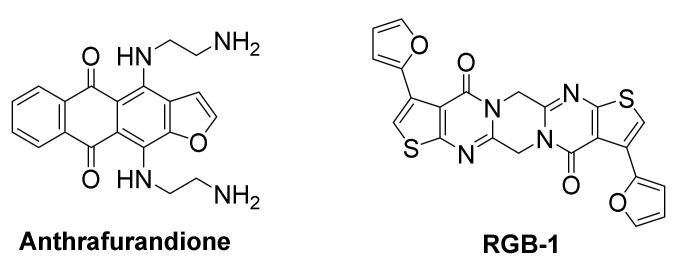
Structures of mRNA G-quadruplex-targeted ligands.

Stabilization of G-quadruplexes in 5’-UTRs of mRNAs or in G-rich sequences within the coding regions of mRNAs has been shown to downregulate and alter protein generation by the ribosome. Recent examples of such ligands (Figure 11) are described below.

#### 3.4.1. KRAS mRNA

The human KRAS transcript contains a 5’-UTR G-rich sequence, capable of forming several stable RNA G-quadruplex structures. A biotin-streptavidin pull-down assay identified an anthrafurandione compound, as a potent binder for KRAS transcript 5’-UTR G-quadruplexes under low-abundance cellular conditions. This ligand represses translation of the mRNA in a dose-dependent manner [165]. In PANC-1 pancreatic cancer cells, this ligand demonstrated high cellular uptake and was found to reduce p21KRAS GTPase to <10% of the control. This downregulation of KRAS triggers apoptosis, accompanied by significant reduction in cell growth and colony formation.

#### 3.4.2. TERRA & NRAS mRNA

The screening of a chemical library comprising 8000 compounds against the TERRA (telomeric repeat containing RNA sequence) led to the identification of a potent ligand, RGB-1, with high binding affinity for TERRA RNA G-quadruplexes, but low affinity for DNA G-quadruplexes, duplex DNA or other nucleic acid secondary structures [166]. The initial success of RGB-1 in repressing translation of TERRA, incorporated in mRNA at the 5’-UTR upstream of a firefly luciferase gene, both in a cell-free system and in HEK293 living cells, in a dose-dependent manner, indicated its potential as protein translation regulator. RGB-1 was then evaluated in MCF-7 cancer cells for translation inhibition of the endogenous oncogenic NRAS mRNA, which contains a G-quadruplex-forming sequence in its 5’-UTR. It also exhibited a dose-dependent reduction of NRAS protein expression, while not affecting protein levels of actin and GAPDH significantly, suggesting this was a RNA G-quadruplex-mediated effect. Further in vitro studies suggested that RGB-1 binding causes stabilization of NRAS G-quadruplex, downregulates NRAS protein expression and may even exploit a novel G-quadruplex-forming site for binding, which was not previously known.

### 3.5. Ligands with Multiple Reported G-Quadruplex Targets

**Figure 12:**
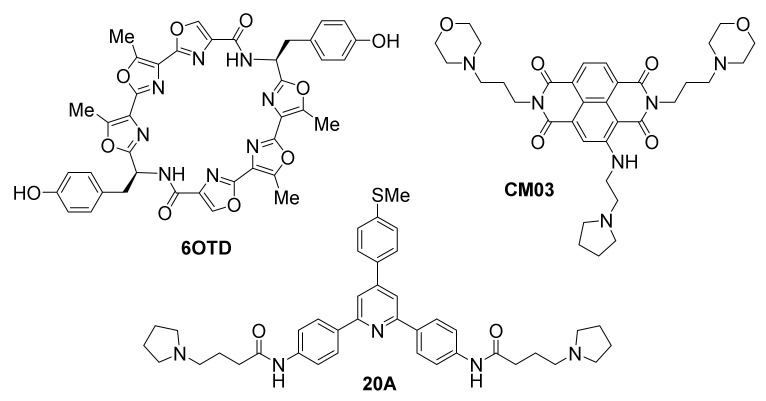
Structures of ligands proposed to target multiple G-quadruplexes.

Figure 12 highlights ligands with putative action against multiple G-quadruplex targets, based on the analysis and interpretation of their observed results.

A recently reported synthetic oxazole/telomestatin derivative, 6OTD, exhibits a promising profile in targeting glioblastoma and glioma stem cells (GSCs) in vitro and in vivo [167]. Developed after the rare natural product telomestatin, one of the most potent stabilizers of telomeric G-quadruplexes known to date [168,169], which the same authors have also evaluated against GSCs [170], 6OTD is crucially more chemically stable and available in gram-scale amounts compared to the natural product, as well as more water-soluble, thus facilitating its application. 6OTD exhibits significant antitumor activity, based on results from a human cancer cell line panel and mouse xenografts [167]. Specifically, 6OTD inhibits growth of glioma stem cells (GSCs) more potently than that of differentiated non-stem glioma cells (NSGCs). DNA damage, G1 cell cycle arrest and apoptosis are observed in the case of GSCs but not NSGCs. DNA damage foci co-localize with telomeres that contain tandem G-quadruplexes, proposed to be stabilized by the ligand, triggering DNA damage stress response selectively in GSCs. The mechanism of action of 6OTD is proposed to be multimodal and involve stabilization of oncogene promoter G-quadruplexes in addition to telomeric G-quadruplexes, since effects on the telomeres alone cannot account for the higher efficiency compared to telomestatin. Evidence of *MYB* G-quadruplex effects are reported. In a mouse xenograft model, 6OTD successfully supppresses intracranial growth of GSC-derived tumors.

CM03 [171], a trisubstituted naphthalene diimide that was designed computationally prior to its synthesis, based on the binding mode of previously known tetrasubstituted diimide MM41 [172], has been found in vitro to cause stabilization of G-quadruplexes from the telomere and from oncogene promoters of *HSP90*, *BCL2* and *KRAS*, but not the duplex control (T-loop) DNA. Its binding affinity to the telomeric G-quadruplex was found to be in the nanomolar range [171]. CM03 acts as a potent cell growth inhibitor of pancreatic ductal adenocarcinoma (PDAC) cell lines in vitro, with in vivo anticancer activity in PDAC animal models, superior to that of the known anticancer drug gemcitabine. The impact of CM03 treatment on global gene expression has been studied by applying whole transcriptome RNA-seq methodology. This approach has revealed a systematic downregulation of multiple genes, rich in putative G-quadruplex-forming sequences, which are involved in cancer pathways of PDAC survival, metastasis and acquisition of drug resistance (such as axon guidance, hippo, mTOR, VEGF, insulin resistance, Rap1 and MAPK signaling pathways). Treatment of asynchronous PANC-1 cells with CM03 resulted in a time-dependent significant increase in BG4 foci, indicative of G-quadruplex stabilization at various sites, as well as induction of DNA damage and replicative stress. These findings suggest a multi-G-quadruplex-mediated mechanism of action against a very hard-to-treat human cancer.

Triarylpyridine 20A is another example of ligand shown to affect multiple G-quadruplexes. It was originally demonstrated that 20A has good affinity and high selectivity for the G-quadruplexes from the telomere and oncogene promoters *KRAS* and *KIT2* [173]. Its NMR structure bound to a telomeric G-quadruplex has been solved [174]. A more recent investigation has shown 20A to cause growth inhibition of cancer cells in culture, in a dose-dependent manner, and in vivo in HeLa mouse xenograft models [175]. This was associated with (p53-independent) induction of senescence and apoptosis. Whole-transcriptome analysis of cells exposed to 20A reveals effects on multiple cellular pathways related to cell growth, DNA damage and ATM and autophagy pathways activation. Overall, >600 genes are reported to be either upregulated or suppressed. Suppressed genes were found to be associated with G-quadruplex-forming motifs in loci other than telomeric regions, particularly upstream of gene transcription termination sites, suggestive of multi-G-quadruplex-mediated transcription-affecting actions. Interestingly, while global DNA damage response was promoted by 20A, telomeric damage was not detected. Disruption of ATM or autophagy pathways sensitizes cells toward apoptosis, suggesting ATM plays the role of linchpin between senescence and cell death.

## 4. Challenges for G-Quadruplex Ligand Progression to Clinical Trials and the Way Forward

The discovery of G-quadruplexes, first in the telomere region, subsequently in oncogene promoters and other regions of DNA and more recently in mRNAs, has initiated a discussion around the natural relevance of G-quadruplexes and has helped unfold a new and exciting field of scientific discovery. While the implication of G-quadruplexes in key biological processes has only recently begun to be widely recognized, it is becoming clear that potential pharmaceutical intervention may be achieved, by means of employing G-quadruplex-targeted ligands [34,35,36]. Such compounds are viewed as potential tools for exploiting G-quadruplex-forming elements in the genome and inducing their transition to fully folded G-quadruplex structures, resulting in anticancer effect(s). The profound impact of G-quadruplexes on regulation of replication, transcription and translation, as well as genome stability and chromatin remodeling, has resulted in their emergence as novel and diverse targets for anticancer research [31,32,33]. Some have expressed the opinion that the interest developed around G-quadruplexes, may even signify the beginning of a new era for DNA-targeted therapeutics [35]. Particular advantages of this approach would be the targeting of sequences associated with ‘gain-of-function’ in cancer, either due to mutations or to existence of multiple copies-related amplification of a gene, as well as the targeting of sequences whose protein product is considered non-druggable (e.g., *MYC*) [115]. In particular, G-quadruplex targets that are unique or prevalent to cancer cells but not normal cells (e.g., *hTERT*), are ideal candidates for modulation.

Undeniably, G-quadruplexes offer untapped potential for the development of novel anticancer therapeutics. There is now a pressing expectation on part of the scientific community for identifying G-quadruplex ligands with efficacy against G-quadruplexes and with appropriate drug-like features, that render them exploitable from a pharmaceutical perspective. However, despite a plethora of G-quadruplex-interacting ligands described in the literature over the last 2 decades [36,37,38,39,40,41,42], to this date only a limited number have entered clinical trials, and none has yet made it through the drug development pipeline.

This lag time may be attributed, in part, to the initial lack of structural information on ligand-G-quadruplex interaction and limited knowledge on ligand operational modes of binding. In the absence of these, rational ligand design was mostly empirical, and modes of binding were putative, based on indirect spectroscopic methods for evaluation of the interaction with the target. To a certain extent, this problem can now be circumvented, due to the publication of several studies describing NMR [174,176] or X-ray structures [177] of complexes between G-quadruplexes and ligands. The availability of this data, in combination with versatile computational approaches, now help inform our design of new-generation ligands with optimized binding capabilities and pre-determined modes of binding.

Other practical challenges, inherent to the problem at hand, also had to be addressed in designing new ligands, such as combining high affinity toward the intended G-quadruplex target with substantial selectivity in favor of that target but against other (potentially competitive) targets, including nucleic acids that may be more abundant in a cellular context. In the direction of achieving high G-quadruplex affinity, the ‘golden rule’ of employing an extended, planar, polyaromatic surface as the ligand’s central scaffold, seemed to be an acceptable solution in a majority of studies, due to the ability of such a scaffold to engage in π-π stacking interactions with the solvent-exposed G-quartets (referred to as ‘end-stacking’), a common feature of all G-quadruplexes [35,36]. Incorporation of heteroatoms in the scaffold or appendage of electron-withdrawing substituents generally led to strong ‘end-stacking’ interactions. The large dimensions of the scaffolds used in G-quadruplex-targeted ligands, especially if they were comparable with the dimensions of a G-quartet, but bulkier than known intercalation agents for duplex DNA, were part of the solution for creating bias in favor of G-quadruplexes and avoiding unwanted targeting to dsDNA. Further enhancement of affinity was achieved by introducing cationic or hydrogen bond-capable side-chains around the periphery of the main scaffold [40]. The exact number and positioning of these side chains often proves critical for selectivity, due to the way these are ‘displayed’ toward other G-quadruplex interaction loci surrounding the G-quartet central core, namely the loops and grooves of the G-quadruplex.

However, in rational design, a persistent challenge for the in cellulo and in vivo application of G-quadruplex ligands still remains: their ability to discriminate between diverse G-quadruplexes. This is due to the fact that a main element of interaction, the exposed G-quartets, are present in all G-quadruplexes. Apart from ‘decorating’ G-quartet-interacting scaffolds with peripheral elements capable of interacting with discriminating features of G-quadruplexes, modern approaches propose turning toward rotationally-flexible oligo-heteroaryl ligands that exclusively target the groove and loop components of G-quadruplexes, rather than G-quartets [151,178,179,180,181]. This alternative approach, especially when reinforced by computational predictions, is likely to reach even higher selectivity levels.

Interestingly, another school of thought advocates in favor of adopting a ‘one-drug-multiple-target approach’ for G-quadruplex ligands, in analogy to the exploitation of multiple kinases as targets for anticancer agents with broad applicability [36]. This notion recognizes the benefits resulting from using a single ligand capable of ‘hitting’ multiple G-quadruplex targets, many of which may induce anticancer consequences, while at the same time implying that absolute G-quadruplex targeting specificity may be nearly impossible to achieve. This approach, which already numbers several successful examples (see Section 3.5), is expected to allow progression of many ‘broad selectivity’ ligands to clinical trials in the following years.

Design principles of G-quadruplex-targeted ligands may be combined today with in silico virtual screening to identify appropriate ligands before actually synthesizing them [171] or may inform the high-throughput synthesis of compound libraries, intended for systematic evaluation against a given G-quadruplex target. Both approaches are expected to increase chances of identifying improved ligand structures, in terms of binding affinity and selectivity, while limiting laborious empirical efforts. In addition to these, the application of target-guided building block selection for G-quadruplex ligand construction [122] and of high-throughput microarrays for selection of strongly- and selectively-interacting ligands [121,165,166], are very likely to enhance success rates.

The requirement for ‘drug-likeness’ of ligands against G-quadruplexes poses an important challenge. Many π-π stacking ligands with propensity for self-aggregation, such as porphyrins, face aqueous solubility issues, a problem that can be by-passed by incorporating in a structure cationic or ionizable groups or ring heteroatoms. Other than that, ligands need to demonstrate satisfactory membrane permeability and cellular uptake, chemical and enzymatic stability, and to abide by Lipinski’s rule of five, to be considered drug-like. Thankfully, the modular character of many ligands designed to interact with G-quadruplexes, readily enables fine-tuning, to make them compliant with these requirements, in advanced stages of drug design. Therefore, newly proposed structures are expected to become more drug-like compared to early examples.

The pharmacokinetic behavior of ligands within cells and within model organisms has not been evaluated systematically and continues to be one of the main bottlenecks for the translation of several potent and selective lead compounds into clinical testing. However, several research efforts worldwide, often involving synergies between academia and industry, have made progress in this sector, and it is hoped that this will continue in the following years, through intensified in cellulo and in vitro screening initiatives.

While significant steps have taken place in ligand design and optimization, the fact that the natural roles of G-quadruplexes are not fully elucidated has previously maintained an uncertainty in proceeding G-quadruplex-targeted lead compounds to in vivo and clinical investigation. Fortunately, over the last few years, with the emergence of various G-quadruplex-specific antibodies [18,19,20,21] and imaging probes [22] that have enabled visualization of G-quadruplexes in live cancer cells, the roles of G-quadruplexes in cancer are being unraveled. These are expected to be further clarified over the next years, thus providing solid background for the development of specialized G-quadruplex modulators and pharmaceuticals. The utilization of whole-transcriptome analyses in modern studies [175] is also expected to further deepen our understanding of the impact of G-quadruplex-targeted ligands—and G-quadruplexes themselves- on cancer-relevant pathways and biological processes. With a focus on prioritizing G-quadruplex targets found to be prevalent in cancer but not normal cells, safer and target-selective drug candidates with limited cytotoxicity to normal cells may be developed. A proof of principle for the therapeutic plausibility of such candidates is provided by the 2 compounds already in clinical trials [157,158], discussed in Section 3.3.

The developments described in this review now generate optimism that the challenges surrounding the medicinal exploitation of G-quadruplexes as a novel fascinating class of anticancer targets are not unsurmountable, and that soon the first examples of clinical compounds against these targets will become available.

## Abbreviations

A:	adenine
A549:	human adenocarcinoma alveolar basal epithelial cells
ATM:	ataxia telangiectasia mutated
ATR:	ataxia and Rad3-related
Bax:	BCL2-associated X protein
*BCL2*:	B-cell lymphoma 2 gene
BG4:	anti-G-quadruplex recombinant antibody
*BRCA*:	breast cancer susceptibility protein-encoding gene
C:	cytocine
CA46:	Burkitt’s lymphoma cancer cells
CDK6:	cyclin-dependent kinase 6
CHK1:	checkpoint kinase 1
CNBP:	cellular nucleic acid-binding protein
Cox-2:	cyclooxygenase-2
CST:	cellular multiprotein complex, composed of proteins CTC1 (copy telomere complex component 1), STN1 (telomere end-binding and capping protein) and TEN1 (telomere length regulation protein) in mammals
3D:	three-dimensional
DOG1:	helicase ATP-binding domain-containing protein
DNA:	deoxyribonucleic acid
DNA pol:	DNA polymerase
dsDNA:	double-stranded DNA
EWS:	Ewing’s sarcoma protein
FANCJ:	Fanconi anemia group J protein (helicase)
FUS:	fused in sarcoma, RNA-binding protein
G:	guanine
GAPDH:	glyceraldehyde 3-phosphate dehydrogenase
GIST:	gastrointestinal stromal tumor
GSC:	glioblastoma/glioma stem cells
GTP:	guanosine triphosphate
HCT15:	colorectal adenocarcinoma cell line
HCT116:	human colon cancer cell line with mutation in codon 13 of the KRAS proto-oncogene
HEK293:	human embryonic kidney cell line
HeLa:	immortal cervical human cell line named after the original donor Henrietta Lacks
Hep-2:	human laryngeal squamous carcinoma cells
HIFA:	hypoxia inducible factor A
HR:	homologous recombination
*HRAS*:	Harvey rat sarcoma viral oncogene homolog
*HSP90*:	heat shock protein 90
*hTERT*:	human telomerase reverse transcriptase gene
IC50:	half maximal inhibitory concentration
IR:	ionizing radiation
Jurkat:	immortalized line of human T lymphocyte cells
*KIT*:	proto-oncogene encoding for receptor tyrosine kinase KIT
*KRAS*:	Kirsten rat sarcoma virus proto-oncogene
MAPK:	mitogen-activated protein kinase
Max:	MYC-associated factor X
MCF-7:	human breast cancer cell line
Mia PaCa 2:	human pancreatic cancer cell line
mRNA:	messenger RNA
mTOR:	mammalian target of rapamycin
*MYB*:	Avian virus myeloblastosis gene homolog, proto-oncogene
*MYC*:	Avian virus myelocytomatosis gene homolog, proto-oncogene
NCT:	United States patent number
NHE III_1_:	nuclease hypersensitive element III_1_
NMR:	nuclear magnetic resonance
*NRAS*:	neuroblastoma rat sarcoma viral gene homolog
NSGC:	non-stem glioma cells
p21:	cyclin-dependent kinase inhibitor 1
p53:	tumor suppressor protein 53
PANC-1:	human pancreatic cancer cell line isolated from ductal pancreatic carcinoma
PARP:	poly(ADP-ribose) polymerase
PCR:	polymerase chain reaction
PDAC:	pancreatic ductal adenocarcinoma
PDGFA:	platelet-derived growth factor subunit A
PIF1:	5’-to-3’ DNA helicase
POT1:	protection-of-telomeres protein 1
*RAD51*:	repair-of-DNA protein-encoding gene
Rap1:	Ras-proximate protein1
*Rb1*:	retinoblastoma gene
rDNA:	ribosomal DNA
*RET*:	rearranged-during-transfection proto-oncogene
RNA:	ribonucleic acid
RNA pol:	RNA polymerase
RNA-seq:	RNA sequencing
rRNA:	ribosomal RNA
RTEL1:	regulator of telomere elongation helicase 1
Saos-2:	human osteosarcoma cancer cell line
SL1:	selective factor 1
SP1:	specificity protein 1
SPR:	surface plasmon resonance
ssDNA:	single-stranded DNA
SW620:	human colon adenocarcinoma cell line
T:	thymine
TERRA:	telomeric repeat-containing RNA
TF:	transcription factor
TPP1:	tripeptidyl-peptidase 1
TRF2:	telomeric repeat-binding factor 2
UTR:	untranslated regions
*VEGF*:	vascular endothelial growth factor gene
